# Gating of tactile information through gamma band during passive arm movement in awake primates

**DOI:** 10.3389/fncir.2015.00064

**Published:** 2015-10-26

**Authors:** Weiguo Song, Joseph T. Francis

**Affiliations:** Department of Physiology and Pharmacology, SUNY Downstate Medical CenterBrooklyn, NY, USA

**Keywords:** somatosensory cortex, thalamus, sensory gating, time-frequency representation, Granger causality

## Abstract

To make precise and prompt action in a dynamic environment, the sensorimotor system needs to integrate all related information. The inflow of somatosensory information to the cerebral cortex is regulated and mostly suppressed by movement, which is commonly referred to as sensory gating or gating. Sensory gating plays an important role in preventing redundant information from reaching the cortex, which should be considered when designing somatosensory neuroprosthetics. Gating can occur at several levels within the sensorimotor pathway, while the underlying mechanism is not yet fully understood. The average sensory evoked potential is commonly used to assess sensory information processing, however the assumption of a stereotyped response to each stimulus is still an open question. Event related spectral perturbation (ERSP), which is the power spectrum after time-frequency decomposition on single trial evoked potentials (total power), could overcome this limitation of averaging and provide additional information for understanding the underlying mechanism. To this aim, neural activities in primary somatosensory cortex (S1), primary motor cortex (M1), and ventral posterolateral (VPL) nucleus of thalamus were recorded simultaneously in two areas (S1 and M1 or S1 and VPL) during passive arm movement and rest in awake monkeys. Our results showed that neural activity at different recording areas demonstrated specific and unique response frequency characteristics. Tactile input induced early high frequency responses followed by low frequency oscillations within sensorimotor circuits, and passive movement suppressed these oscillations either in a phase-locked or non-phase-locked manner. Sensory gating by movement was non-phase-locked in M1, and complex in sensory areas. VPL showed gating of non-phase-locked at gamma band and mix of phase-locked and non-phase-locked at low frequency, while S1 showed gating of phase-locked and non-phase-locked at gamma band and an early phase-locked elevation followed by non-phase-locked gating at low frequency. Granger causality (GC) analysis showed bidirectional coupling between VPL and S1, while GC between M1 and S1 was not responsive to tactile input. Thus, these results suggest that tactile input is dominantly transmitted along the ascending direction from VPL to S1, and the sensory input is suppressed during movement through a bottom-up strategy within the gamma-band during passive movement.

## Introduction

Understanding how sensory information is processed in dynamic environments will provide important basic information on neural encoding and for designing realistic sensory prosthetics, such as when, where and how to provide effective sensory feedback without affecting the ongoing action. In recent years the study of, and production of brain machine interfaces (BMIs), has become popular in biomedical engineering. The study of BMIs has also lead to some interesting basic neuroscience research (Jackson et al., 2006; Ganguly and Carmena, 2009; Marsh et al., 2015). Initially most BMIs decoded intention, however increasing efforts are being put into neuroprosthetics that stimulate the brain directly, such as toward repair of damaged neural systems (Kerr et al., 2012; Li et al., 2015), or as sensory inputs (Brockmeier et al., 2011; Li et al., 2013, 2014; Tabot et al., 2014). Our overall strategy on this last front has been to recreate cortical neural responses to touch by directly stimulating the VPL thalamus or somatosensory cortex. Thus, knowing how passive and active movements change the neural representation will become key for our system to produce the appropriate cortical neural response under these different types of movement. Toward this goal we present here work from passive movement.

Sensory inputs are initiated from peripheral receptor and transmitted through the spinal cord via thalamus to cortex, and sensory information could be regulated at each of these different levels during behavior. Movement could activate peripheral sensory receptors that activate neurons along the somatosensory pathway, however sensory information is commonly suppressed during movement (Chapin and Woodward, 1982; Chapman et al., 1988; Jiang et al., 1991; Urbain and Deschênes, 2007; Song and Francis, 2013), during the preparatory period before movement onset (Nelson et al., 1991; Ogata et al., 2009; Seki and Fetz, 2012), and even during observation of movement (Voisin et al., 2011). Sensory gating can occur at spinal (Ghez and Pisa, 1972; Seki and Fetz, 2012), brainstem (Furuta et al., 2008), and thalamic levels (Aguilar and Castro-Alamancos, 2005; Lavallée et al., 2005; Urbain and Deschênes, 2007), and is stronger during active movement than passive movement (London and Miller, 2012; Seki and Fetz, 2012).

Sensory evoked potentials (SEPs), which are thought to represent postsynaptic potentials from cells in the vicinity of the recording electrodes, are commonly used for evaluation of sensory information processing (Starr and Cohen, 1985; Seki and Fetz, 2012). Traditional SEP calculations are based on the assumption that a stereotyped pattern of phase-locked electrical activity is superimposed onto an independent stationary stochastic process, which is canceled out during averaging. However, the amplitude and latency of the evoked potential are not constant across trials and may depend on the ongoing activity and carry information (Scaglione et al., 2011). To overcome this limitation, event related spectral perturbation (ERSP) analysis was designed by applying time-frequency decomposition to single trials before averaging (Delorme and Makeig, 2004). By calculating the spectral power from either single trial evoked potentials or trial averaged SEP, effects from phase-locked and non-phase-locked responses may be teased out (Tallon-Baudry et al., 1996). It has been indicated that phase-locked and non-phase-locked responses arise from different sources (Kalcher and Pfurtscheller, 1995; David et al., 2006), thus the dissection of each contribution may help to better understand the mechanism of sensory gating.

Most findings about sensory gating have been based on individual neural structures or from stand-alone observations (Starr and Cohen, 1985; Marlinski et al., 2012; Seki and Fetz, 2012). As there are extensive anatomical and functional interconnections between and within somatosensory areas (Deschênes et al., 1998; Hunnicutt et al., 2014; Kinnischtzke et al., 2014), some of the changes triggered in one region may influence changes in other regions. However, it is still unclear how movement modulates the interactions between regions distributed across sensory-motor circuits and how sensory information is processed by the intrinsic circuit without a behavioral task.

Granger causality (GC) provides an efficient way to probe the causal/directional coupling between two signals, and has been used to test the interactions between two brain structures (Brovelli et al., 2004). To this aim, local field potentials (LFPs) were recorded simultaneously from microelectrode arrays implanted in cortical areas (S1, M1) and the VPL. Neuronal oscillatory activity in each area was assessed after time-frequency decomposition on single trial evoked responses and trial averaged SEP, and the functional connections between areas were assessed by using GC.

## Materials and methods

Four rhesus monkeys (A, male 4.1 Kg; J, male 3.9 Kg; K, female 3.7 Kg; N, male 5.3 Kg) were used in these experiments. Care and treatment of the animals during all stages of the experiments were approved by the Division of Laboratory Animal Resources and Institutional Animal Care and Use Committee of SUNY Downstate Medical Center.

### Surgical procedure

**Figure 1:**
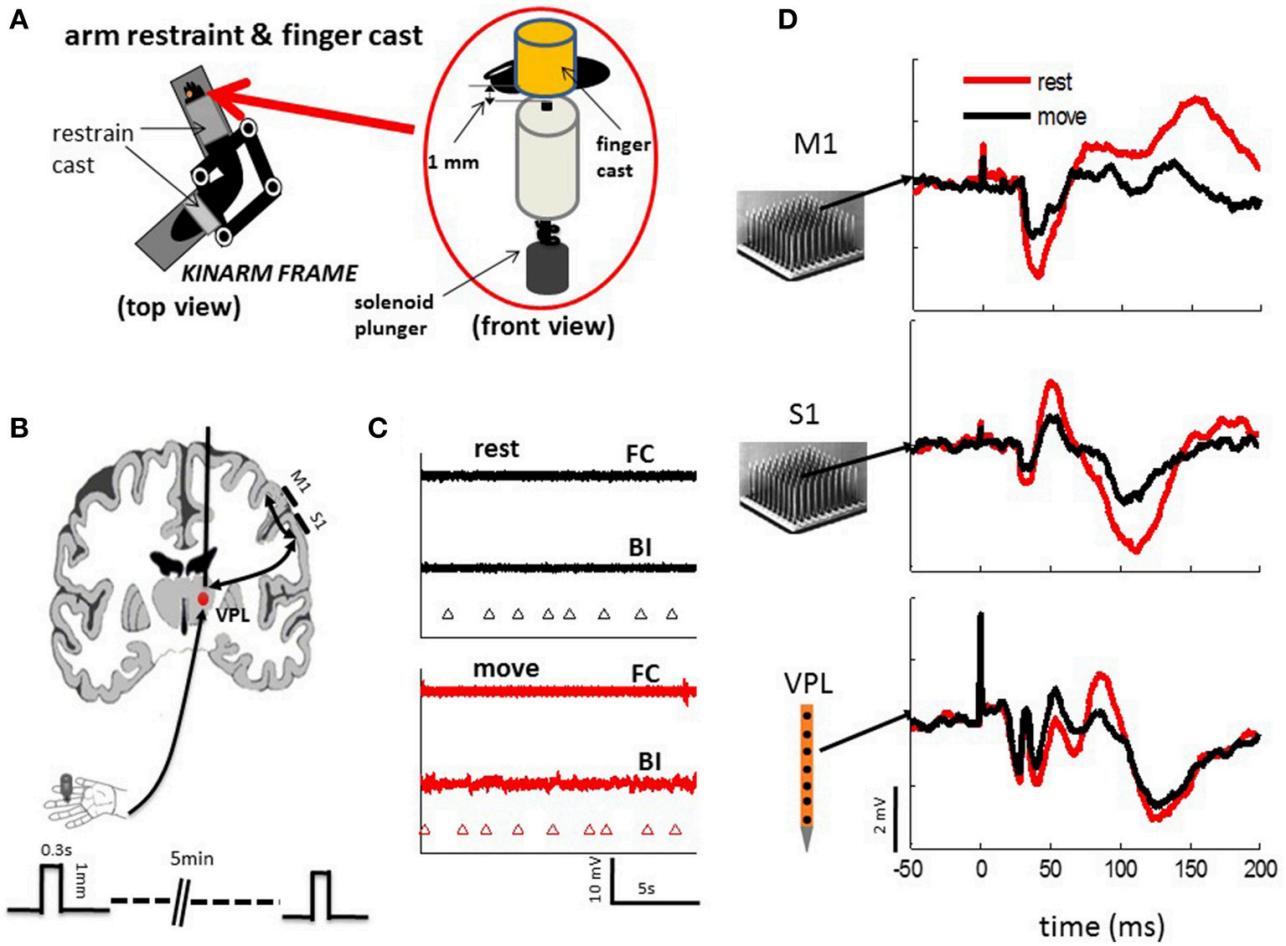
**Experimental setups**. **(A)** Monkeys were seated comfortably in a non-human primate chair with their right arm and hand restrained on a KINARM (BKIN Technologies), which allows free movement of the arm in the horizontal plane. The fingers are held in place by cast that allows tactile stimuli to be delivered reliably from a solenoid plunger under both the resting condition and passive movement of the arm. **(B)** Neuronal activities were recorded from electrode arrays in M1 and S1 (10 by 10, Blackrock Microsystems) or S1 and VPL (24 sites linear array, Microprobes Com) during tactile stimulation to the finger pad. Three-hundred to six-hundred stimuli were randomly delivered via the tactile stimulator **(A)** at a mean frequency of 0.5 Hz under resting and passive arm movement. Each tactile stimulus (one stroke of solenoid) was applied for 0.3 s on the skin, which indents the finger pad of either index, middle, or ring finger by 1 mm. **(C)** EMG activities from the major muscle were (BI, biceps; FC, flexor carpi) recorded during rest and movement. There were no obvious active movements observed during either rest or passive movement. Triangle marker indicate the time of tactile stimulation. **(D)** Example evoked responses from a single channel of each array in M1, S1, and VPL show from typical session.

Following our detailed procedure for head-post and electrode array implantation (Chhatbar et al., 2010), 96-Platinum-Iridum microelectrode arrays (10 by 10; electrode pitch 400 um and electrode length 1.0 or 1.5 mm; Blackrock Microsystems) were pneumatically inserted in left S1/M1 hand representation area, which demonstrated clear response from a sharp probing electrode during touching the right fingers. The M1 and S1 arrays were placed on the bank of pre and post-central sulcus, respectively (Figure 1B). To implant the deep VPL array, MRI images were acquired with 3T Siemens scanner before surgery, and were registered onto the atlas of a standard rhesus brain (Frey et al., 2011). With the guidance of the image and the stereotaxic coordination, a 24-channel linear array (LMA, Microprobes Inc.) was inserted in the VPL nucleus of thalamus on the left hemisphere (monkey N; Figure 1B). Similar to S1 implantation, the implantation location of VPL array was further confirmed during electrode insertion by using the receptive field mapping technique.

### Neural recording

Recordings began 3 weeks after implantation surgery. Neuronal activities were acquired through unity gain head-stages (Plexon Inc.) with a multichannel acquisition processor (MAP, Plexon Inc.). LFPs from different recording areas were acquired simultaneously (M1 and S1 or S1 and VPL) (see example in Figure 1D). LFPs were amplified (gain 500–1000), filtered (0.3–200 Hz) and digitized at a sampling frequency of 1 or 2 kHz. Up to 32 channels (every three channel on the 96 array) of LFP in M1 and S1, and up to 23 channels in VPL were acquired. Power line noise (60 Hz) was removed with offline notch filter (iirnotch, Matlab). Previously we reported unit activities in S1 from monkeys A and N (Song and Francis, 2013), while this paper uses different datasets with LFP recordings.

### Testing protocols

The testing procedure was reported previously (Song et al., 2013). In short, monkeys were seated quietly in a primate chair with their right arms restrained to the KINARM exoskeletal robotic system (BKIN Technologies), and the fingers to be tactile stimulated were put in a finger cast, which was modified by drilling a hole through a cylinder plastic tube. A plunger was attached to the bottom side. Then identical tactile stimuli were randomly delivered by indenting a finger pad (around 1 mm in depth; 0.2–0.3 s duration; 0.5 Hz mean frequency) with a solenoid actuator (plunger diameter: 1 mm; STA-195201, Ladex Inc.; see Figure 1A), which was controlled by a PC via a digital card (PCI-6229, National Instruments Inc.). During tactile stimulation the KINARM was either locked in place (rest) or moved slowly and smoothly by an experimenter within the horizontal plane (30 by 40 cm). Sessions of active arm movement were excluded, as they could be felt by the experimenter and validated with electromyography recordings from a few major forearm muscles (Figure 1C). Each daily training session consisted of several 5-min tactile stimulation epochs (300–600 epochs each session) of either resting or passively moving. In this paper, only the recordings from the left sides of M1, S1 and VPL, which are contralateral to the tested right hand, are present.

### Data analysis

#### Time-frequency representation

**Figure 2:**
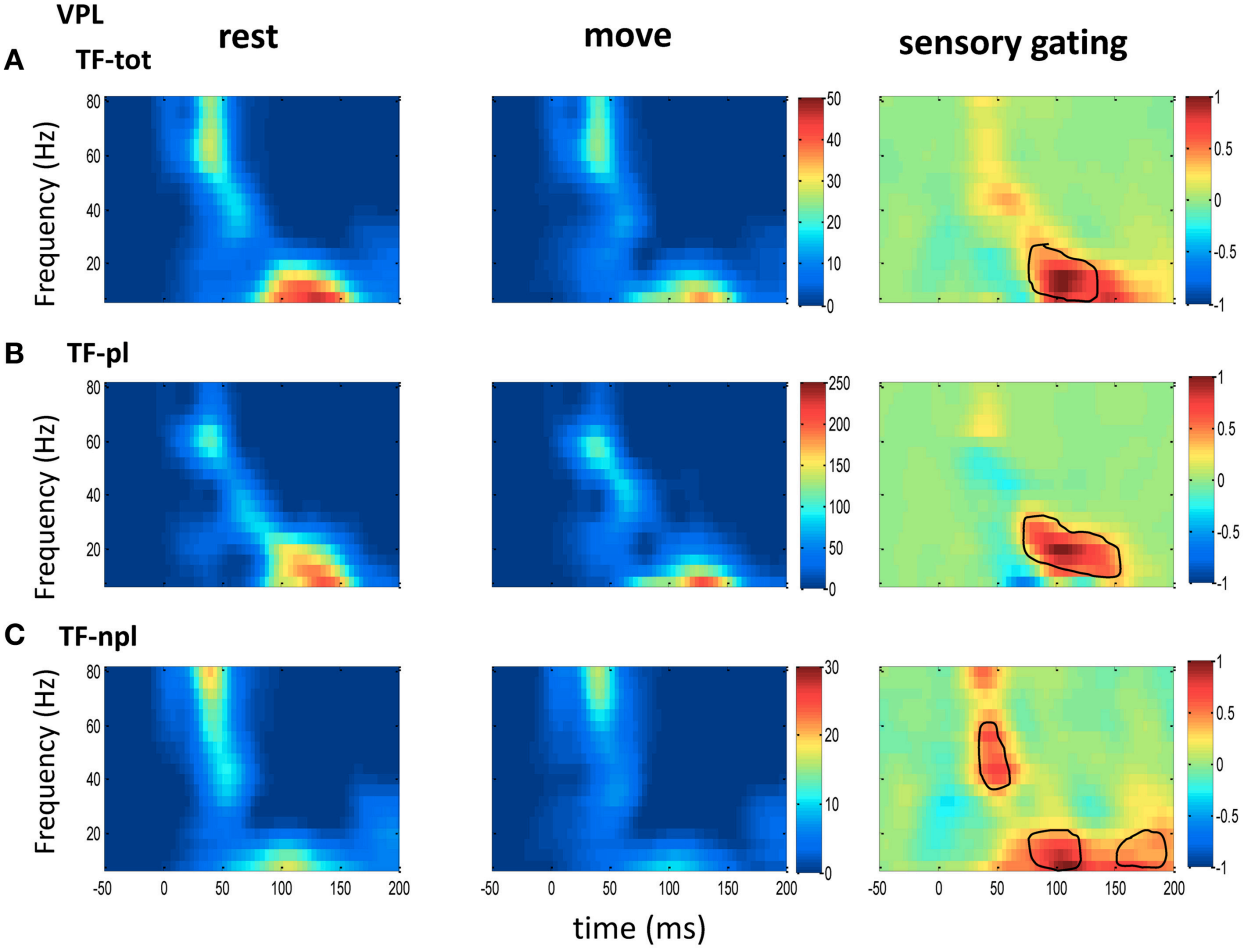
**The grand average powers in VPL**. **(A)** Total powers during rest (left) and move (right) show early high gamma oscillations followed by low frequency oscillation. Movement induced sensory gating (right: rest–move) occurs at low frequency bands (right). **(B)** The phase-locked power, which is time-frequency representation on trial-averaged SEPs, shows similar pattern to that of total power but shifted toward low frequency band. **(C)** The non-phase-locked power shows stronger oscillation at gamma band than at low frequency band, and sensory gating presents at both gamma band and low frequency band. Colorbar in each map of rest and move condition represents normalized power (std of baseline); Colorbar of the sensory gating map was normalized to maximum value of the map, and elements within circle represent significantly changed (*p* < 0.05 for signrank test, *n* = 10).

**Figure 3:**
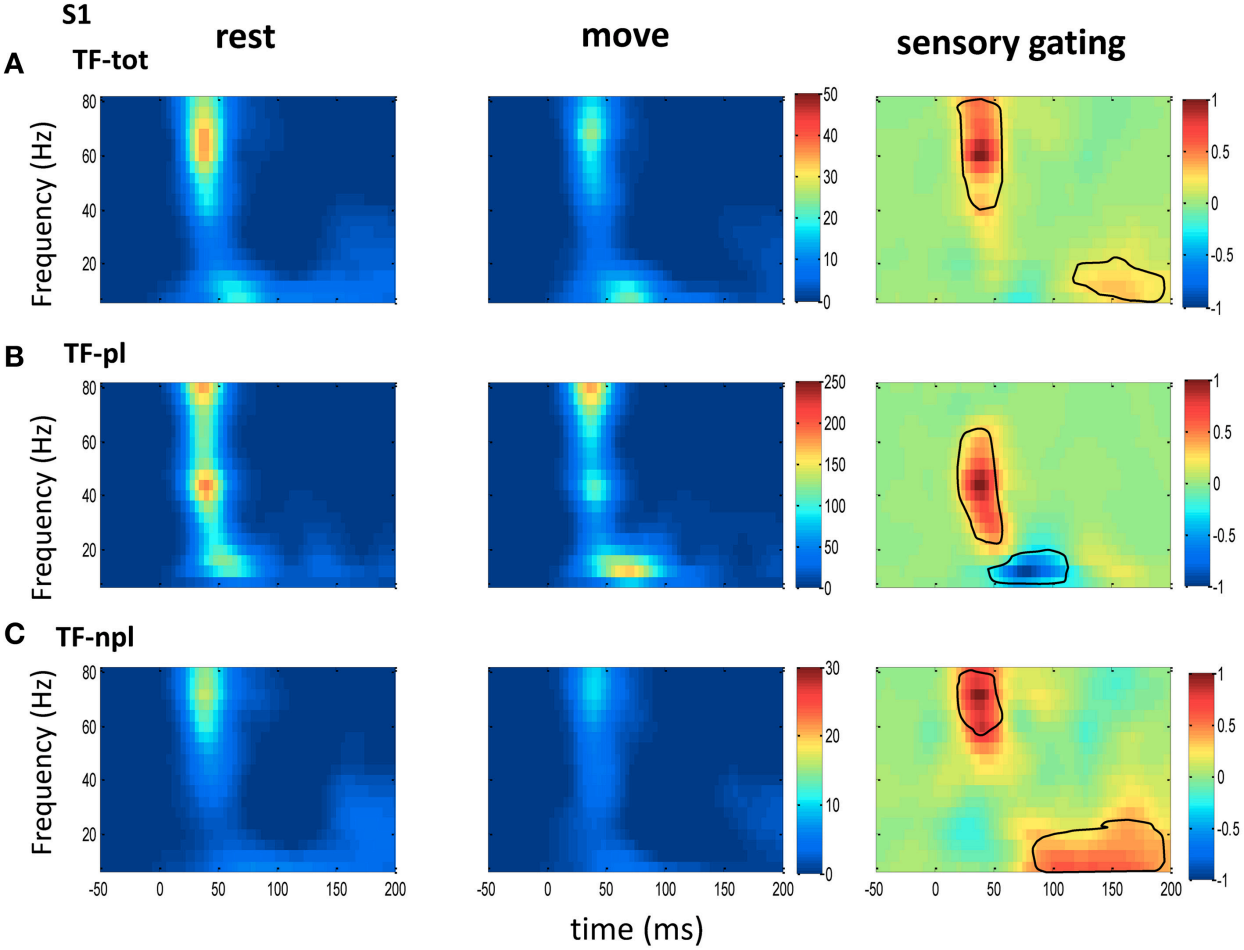
**The grand average powers in S1**. **(A)** Total powers during rest (left) and move (right) show early high gamma oscillations followed by weak low frequency oscillations, which starts earlier than that in VPL (Figure 2). Movement induced sensory gating (right) is stronger at high gamma band than later low frequency band. **(B)** Compared to that of total power, the phase-locked power shows low frequency shifted pattern, and the sensory gating only at low gamma band while an elevation at low frequency band. **(C)** Non-phase-locked power shows dominant high frequency oscillation, and sensory gating presents strong early high gamma band and weak late low frequency band. The same convention of the colorbar and normalization is used in each map as in Figure 2 (*p* < 0.05 for signrank test, *n* = 26).

**Figure 4:**
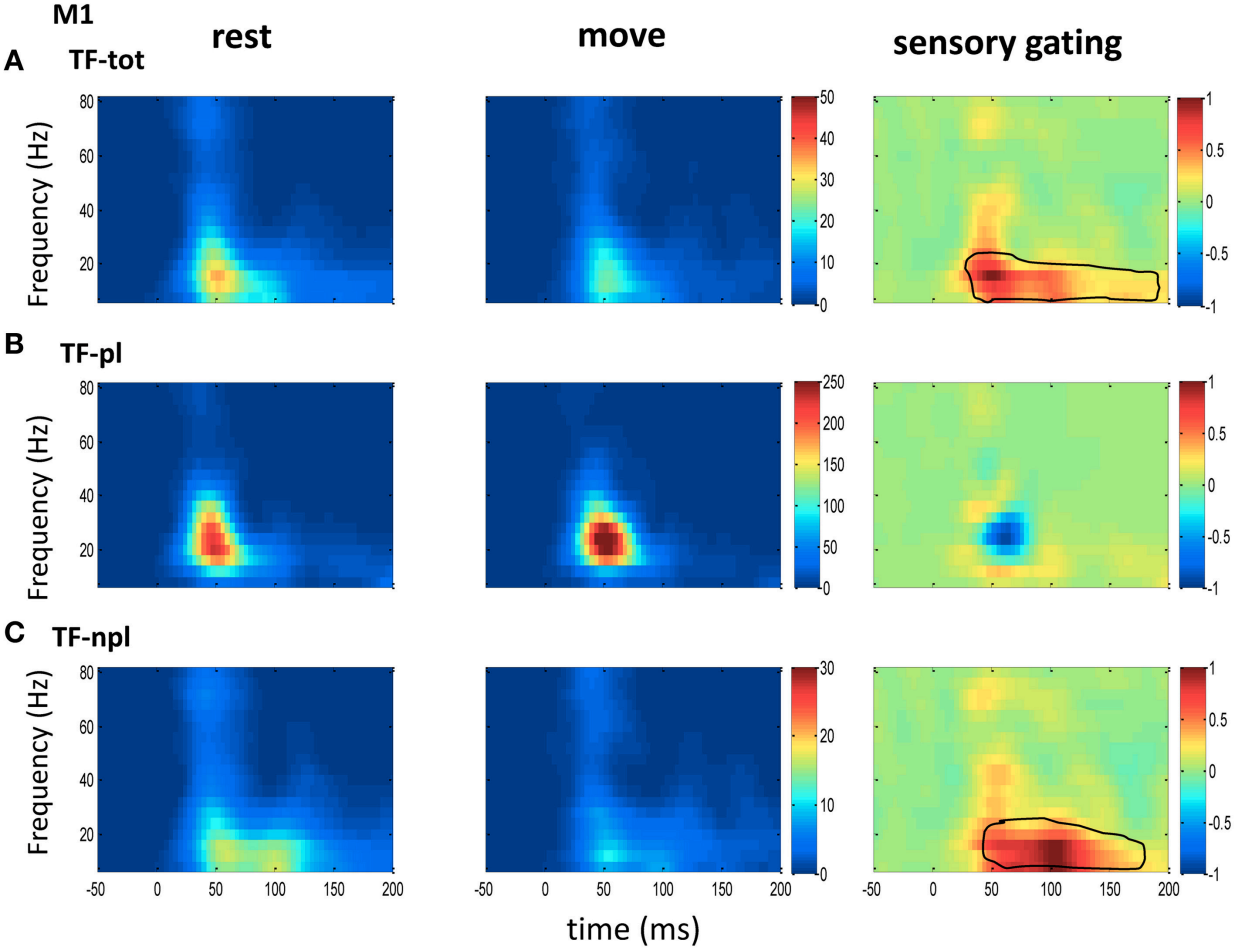
**The grand average powers in M1**. **(A)** Different from the powers in sensory areas (VPL of Figure 2 and S1 of Figure 3), total powers during rest (left) and move (middle) in M1 show oscillatory activities dominantly at low frequency band. Movement induced sensory gating is also at low frequency band, which starts earlier than those in sensory areas (VPL and S1). **(B)** The phase-locked power shows similar pattern to that of total power, but different from the total power, instead of sensory gating, there is a small sensory elevation at low gamma band. **(C)** Compared the non-phase-locked power with sensory areas, the sensory gating in M1 only presents at low frequency band. The same convention of the colorbar and normalization is used in each map as in Figure 2 (*p* < 0.05 for signrank test, *n* = 16).

Neural response epochs (trials), which were defined as from 100 ms before and 200 ms after each tactile stimulus onset, were recorded during tactile stimulation on the finger pads. After rejection of epochs contaminated with artifacts by visually checking the baseline neural activity (100 ms before stimulation onset). SEPs were calculated as the average response to tactile stimuli across all epochs. ERSP was calculated from time-frequency decomposition of single trial response (total power) or trial averaged SEP (phase-locked power; Delorme and Makeig, 2004). We further calculated time-frequency decomposition of the single trail response after SEP was removed, which was termed non-phase-locked power (Cohen and Donner, 2013). The difference between total power, non-phase-locked and phase-locked power could help tease out the underlying contributions from phase-locked and non-phase-locked components (Tallon-Baudry et al., 1996). To obtain optimal time-frequency resolution, we applied wavelet decomposition methods. To further reduce the sensitivity of noisy trials, the ERSP was normalized (subtract mean and divided by standard deviation in each whole trial) before averaging across trials (Grandchamp and Delorme, 2011) and then baseline corrected (subtract mean power of baseline and divided by standard deviation of baseline power. To compare the gating effect across areas, each gating map (rest–move) was normalized to the maximum power in the map. The oscillatory activity was assessed at low frequency (beta-band: 14–30 Hz) and gamma-band (30–80 Hz). Then the power spectrum was averaged across sessions to give a grand average (see Figures 2–**4**).

#### Granger causality

GC has been used to research the temporal interactions between brain areas (Brovelli et al., 2004). To study the causal relation between two signals, the time series of these signals are first modeled with bivariate autoregressive process as

(1) $$x\left(t\right)=\sum_{i\;=\;1}^{N}a_{11,i}x(t-i)+\sum_{i\;=\;1}^{N}a_{12,i}y(t-i)+E_{1}(t),$$

(2) $$y\left(t\right)=\sum_{i\;=\;1}^{N}a_{21,i}x(t-i)+\sum_{i\;=\;1}^{N}a_{22,i}y(t-i)+E_{2}\left(t\right),$$

where *N* is the order of the autoregressive model, *a*_11, *i*_, *a*_12, *i*_, *a*_21, *i*_ *and a*_22, *i*_, are the regression coefficients, and *E*_1_(*t*) *and E*_2_(*t*) are the predication error with covariance matrix $\Sigma =\left(\begin{array}{cc} \Sigma_{11}, & \Sigma_{12}\\ \Sigma_{21}, & \Sigma_{22} \end{array}\right)$. Then Equations (1) and (2) are transformed into frequency domain

(3) $$\left(\begin{array}{c} X(f)\\ Y(f) \end{array}\right)=H\left(\begin{array}{c} E_{1}(f)\\ E_{2}(f) \end{array}\right),$$

where *H* is the transfer matrix with $H={\left(\begin{array}{cc} A_{11} & A_{12}\\ A_{21} & A_{22} \end{array}\right)}^{-1}.$ Then the spectral matrix of the system can be calculated as

(4) $$S\left(f\right)=<H\left(f\right)\Sigma H^{*}\left(f\right)>$$

where ^*^ corresponds to transposition and complex conjugation of *H*(*f*). Finally, the GC from *y to x* is expressed as

(5) $$GC_{y\to x}\left(f\right)=\;-ln\left|\left(1-\left(\Sigma_{22}-\frac{\Sigma_{12}^{2}}{\Sigma_{11}}\right){\left|H_{12}\left(f\right)\right|}^{2}/S_{11}\left(f\right)\right)\right|,$$

and *x to y* as

(6) $$GC_{x\to y}(f)=\;-ln\left|\left(1-\left(\Sigma_{11}-\frac{\Sigma_{21}^{2}}{\Sigma_{22}}\right)|H_{21}(f){|}^{2}/S_{22}(f)\right)\right|.$$

The LFPs from different channels of each recording array were averaged together before use to yield a low-noise representation to build the model. Following the procedure to calculate GC (Brovelli et al., 2004), the ensemble mean of SEP from each recording area was subtracted point-wise from each epoch time series, and then the amplitude was divided by the temporal standard deviation to give equal weight for different recording areas and epochs. GC between two areas was calculated with the toolbox developed by Seth (Seth, 2010) with spectral autoregressive modeling from BSMART (Cui et al., 2008). The order of the model was chosen based on Akaike information criterion (AIC), which drops monotonically with increased model order. When considering the small decrease in AIC for order higher than 10, a maximum order of 10 was used. A 100 ms window with 4 ms moving step was used in this model. Various lengths of windows and steps were also tested, and the overall results were consistent. The GC at each frequency was normalized to its baseline (100 ms before tactile onset), and represented as ratio change over baseline. To confirm the GC in individual sessions was not from random connections, we cross-validated the GC by using a bootstrap strategy (resampling with replacement from original data while preserving both serial order and causal relations: *n* = 500). The pattern (peaks and latencies at difference frequency bands) of the grand mean map agrees with the pattern in the bootstrapped map over 95% confidence level.

### Statistical analysis

Similar patterns were found across animals, thus all the data from different monkeys was pooled to have a statistical test. Parametric paired or unpaired two sample test (ttest or ttest2, Matlab) was used between different conditions for a normal distribution, and non-parametric rank test (ranksum or signrank, Matlab) was used otherwise. The normality was tested with Jarque-Bera test (jbtest, Matlab). The significance level of each test was set at 0.05, unless stated otherwise. In each sensory gating map, area within a circle represents elements significantly different from zero. Only meaningful area, which was defined as more than 200 elements (equivalent to 20 ms by 10 Hz) connected, was drawn. All analyses were performed using Matlab (MathWorks Inc.).

## Results

Power spectrum in each area and GC between two simultaneously recorded areas (VPL vs. S1 and M1 vs. S1) were analyzed for each session. A total of 26 sessions (10 from VPL and S1; 16 from M1 and S1) of LFP responses to tactile stimulation were recorded in four quiet awake monkeys. There were around 300–600 epochs under both rest and passive arm movement conditions in each session.

### Power spectrum and sensory gating by movement

To understand the mechanism underlying sensory gating, the grand average of total power, non-phase-locked power and phase-locked power were calculated at each recording area (Figures 2–**4**). The total power showed distinct pattern for each area and frequency band, and it was modulated by movement in each area. The total power in sensory areas (VPL and S1) showed short bursts of high frequency oscillations (at 45 ms after tactile input), which were followed by low frequency oscillations. M1 was dominated by low frequency oscillations (at 50 ms after tactile input), which encode movement related information (Rickert et al., 2005). The total power was significantly suppressed by movement at gamma band in S1 and at low frequencies across all areas (sensory gating of Figures 2A, 3A, 4A). While low frequency oscillations were stronger in M1 than in sensory areas (VPL and S1) during both rest and movement. When comparing the gating of phase-locked and non-phase-locked power, the non-phase-locked power was suppressed in high frequency band following tactile input in VPL (Figures 2B,C). In S1, there were immediate suppressions for both phase-locked and non-phase-locked power, while the suppression was in higher frequency band from phase-locked than from non-phase-locked component. Surprisingly, there was a low frequency enhancing in the phase-locked power while suppressing in non-phase-locked power (Figures 3B,C). In M1, the gating was from the non-phase-locked power at low frequency band and no significant change was found in the phase-locked power during movement and rest (Figures 4B,C). In summary, sensory gating was initiated from gamma band in both non-phase-locked oscillations of VPL and S1 and phase-locked oscillation of S1, and then followed by non-phase-locked oscillation in M1 and further a low frequency suppression in VPL and S1.

### Granger-causality and the effect of movement

**Figure 5:**
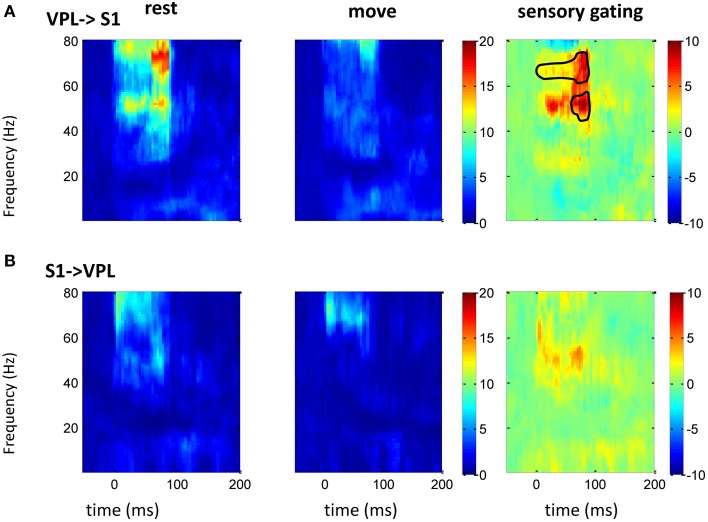
**GC between VPL and S1 in frequency domain**. After normalizing the GC at each frequency to its baseline (100 ms before tactile onset), the time-frequency GC map shows strong directional coupling from VPL to S1 **(A)** than that from S1 to VPL **(B)** during rest (left) and movement condition (middle). The movement induced sensory gating (right), which is the difference of rest and move, is dominantly at gamma frequency band and is stronger and faster from VPL to S1 than that from S1 to VPL. Each heat-map is the mean time-frequency representation across sessions (*n* = 10). Area within circle represents elements significantly different from zero (*p* < 0.05 for signrank test, *n* = 10).

**Figure 6:**
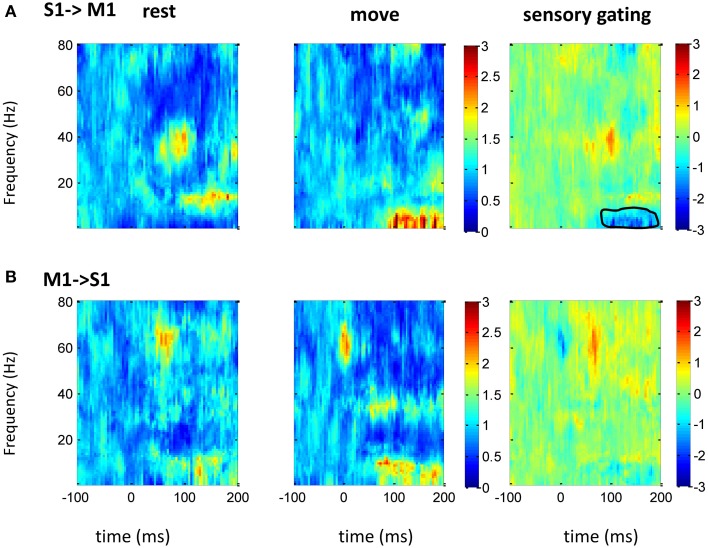
**GC between M1 and S1 in frequency domain**. After normalizing the GC at each frequency to its baseline (100 ms before tactile onset), the time-frequency GC map does not show clear directional coupling pattern from M1 to S1 **(A)** or from S1 to VPL **(B)** during rest (left) and movement condition (middle). Instead of sensory gating, movement significantly increases the GC from S1 to M1 at low frequency band (right). Each heatmap is the mean time-frequency representation across sessions (*n* = 16). The same convention of legend is used as in Figure 5.

The above spectrum analyses showed that neural oscillatory activities were regulated differently by movement at different areas. As there exist anatomical and functional connections between these areas, response at these areas might interact each other. Thus, GC between two areas (*n* = 16 between S1 and M1; *n* = 10 between S1 and VPL) was analyzed. Compared with traditional correlation analysis, GC provides directional information, which helps to resolve the temporal relations between the regions. There was bidirectional GC between VPL and S1, which was modulated by sensory input dominantly at gamma band. As expected, the GC from VPL to S1 was stronger than that from S1 to VPL, and movement suppressed the GC (Figure 5). The suppression of GC was stronger along the ascending direction than descending direction (right of Figure 5). Although both M1 and S1 showed sensory modulated oscillations (Figures 2–4), the GC between S1 and M1 was not strongly modulated by the tactile input. Movement significantly (while weakly compared with GC between VPL and S1) increased the GC at low frequency band from S1 to M1 (Figure 6A), which might originate from the non-phase-locked low frequency oscillation in M1 and S1 (Figures 3, 4). The GC from M1 to S1 was not significantly changed (Figure 6B).

## Discussion

### Phase-locked and non-phase-locked sensory gating show frequency dependency and area specificity

Sensory information is regulated during sensorimotor integration in dynamic environments (Ghez and Pisa, 1972; Chapin and Woodward, 1982; London and Miller, 2013), as well as by attention and cognition (Bollimunta et al., 2011), while the underlying mechanisms are not fully understood. The difference between the total power, the phase-locked power and non-phase-locked power at each area suggests that sensory gating may not only come from the phase-locked stereotyped responses, but is also present in the non-phase-locked ongoing activities. The phase-locked and non-phase-locked responses were thought to reflect different neural processes and represent different underlying neuronal mechanisms (David et al., 2006). Sensory gating in motor cortex was only found in the low frequency of the total power not in the phase-locked power, which shows that the non-phase-locked low frequency oscillation plays an important role during even passive movement. This low frequency oscillation (alpha/beta) is commonly observed in sensorimotor cortex and regulated by movement or attention (Bollimunta et al., 2011; Davis et al., 2012), and it is thought to be an indication of an idling state of the brain, but it may also play a role in sensory-motor integration (Başar et al., 1997; Brovelli et al., 2004). On the other hand, high frequency gamma oscillations have been linked to perception, stimulus specificity and higher-level cognition (Tallon-Baudry et al., 1996; Schaefer et al., 2006; Haegens et al., 2011). S1 showed strong gamma band gating during movement in both the total power and phase-locked power, while the phase-locked power shifted toward low gamma. This is not surprising, as phase-locked power was calculated from trial averaged SEP, which may have canceled out some high frequency “noise.” In line with what has been found in visual and auditory systems (Bertrand and Tallon-Baudry, 2000; Trautner et al., 2006), the gamma oscillation in sensory areas could also be related with tactile representation or stimulus onset through bottom-up mechanisms of feature binding and to enhance sensory transmission (Paik et al., 2009). This putative feature binding ability in S1 was interrupted or suppressed during movement, and it was mostly through non-phase-locked ongoing oscillations, thus further caused the suppression of GC at gamma band (Figure 5). The presence of both high and low frequency gating in S1 might imply multiple neural populations oscillating at different frequencies, which would allow parallel computation of information within the same region (Crone et al., 2001), or the same population from different feedback loops. The gating of the total power in VPL was dominatingly from the phase-locked effect of low frequency band, while there exists non-phase-locked effect at gamma band. This suggests that temporal coding or synchrony could be important during regulation of sensory input in VPL.

### Sensory gating through gamma band along the ascending direction

Sensory gating by movement was observed at different individual areas/levels and under different tasks (Jiang et al., 1991; McCormick and Bal, 1994; Aguilar and Castro-Alamancos, 2005; Urbain and Deschênes, 2007; Furuta et al., 2008; Ogata et al., 2009; Seki and Fetz, 2012), but by using GC we showed that there were only strong directional coupling between sensory areas (S1 vs. VPL). GC along the ascending direction was larger than that along the descending direction. This agrees with the direction of sensory transmission, and corticothalamic projections also demonstrated GC from S1 to VPL. Interestingly, although gamma and low frequency oscillations presented in both VPL and S1, GC was only found in gamma, which indicates that gamma oscillations could bind sensory input across areas. Gamma rhythms are thought to be involved in interregional communication and selection of salient stimuli (Fries, 2009). This frequency dependent sensory information processing was also found recently in the primate visual system, and it was suggested that rhythms of different frequencies act as distinct channels that differentially route top-down and bottom-up signals (Bastos et al., 2015). Movement strongly suppressed directional coupling from VPL to S1, which suggests that sensory gating was from a bottom-up strategy. While the suppression of gamma band power only in S1 not in VPL suggests lateral inhibition might be strongly involved within S1, which also affects sensory processing at VPL (Figure 2). Surprisingly the directional coupling between S1 to M1 did not show significant modulation to tactile stimulation, and there is a weak while significant enhancement at low frequency band. This might come from the non-phase-locked oscillations at S1 and M1 (Figures 3, 4). The stronger sensory information transmission from S1 to M1 during movement than during rest might arise from the activation of the sensory neurons by movement (Fetz et al., 1980; Soso and Fetz, 1980; Cohen et al., 1994). This suggests that the low frequency oscillation did not bind across the network due to sensory input specifically. The non-tactile modulated directional coupling between M1 and S1 could arise from reciprocal connections between S1 and M1 or from common drive to M1 and S1 from thalamus (Asanuma and Fernandez, 1974). But as there was no simultaneous recording between VPL and M1, the direct interaction between them was not testable in this study. It is worth mentioning that compared with the previous movement related active sensing tasks (London and Miller, 2012; Seki and Fetz, 2012), the passive arm movement task had neither an explicitly motivated motor planning phase or active sensing phase nor any reward or cognition involvement, thus it represents intrinsic information processing within the circuit, and the modulation most likely comes from low level sensory information processing. GC between M1 and S1 did not modulate to tactile stimulation further suggesting no high level network involvement in our task, thus our sensory gating was dominantly arising from lower level via a bottom-up mechanism.

In conclusion, tactile stimulation evoked oscillatory activities across the sensorimotor loop, while movement suppressed the oscillation either in a phase-locked or non-phase-locked manner dependent on frequency band and area. Tactile information is dominantly transmitted along the ascending direction from VPL to S1, which is regulated during movement through a bottom-up mechanism within the gamma-band.

### Conflict of interest statement

The authors declare that the research was conducted in the absence of any commercial or financial relationships that could be construed as a potential conflict of interest.
